# T regulatory cells: an overview and intervention techniques to modulate allergy outcome

**DOI:** 10.1186/1476-7961-7-5

**Published:** 2009-03-12

**Authors:** Subhadra Nandakumar, Christopher WT Miller, Uday Kumaraguru

**Affiliations:** 1 Department of Microbiology, College of Medicine, East Tennessee State University, Johnson City, TN-37614, USA; 2 Division of Allergy and Immunology, Department of Internal Medicine, College of Medicine, East Tennessee State University, Johnson City, TN-37614, USA

## Abstract

Dysregulated immune response results in inflammatory symptoms in the respiratory mucosa leading to asthma and allergy in susceptible individuals. The T helper type 2 (Th2) subsets are primarily involved in this disease process. Nevertheless, there is growing evidence in support of T cells with regulatory potential that operates in non-allergic individuals. These regulatory T cells occur naturally are called natural T regulatory cells (nTregs) and express the transcription factor Foxp3. They are selected in the thymus and move to the periphery. The CD4 Th cells in the periphery can be induced to become regulatory T cells and hence called induced or adaptive T regulatory cells. These cells can make IL-10 or TGF-b or both, by which they attain most of their suppressive activity. This review gives an overview of the regulatory T cells, their role in allergic diseases and explores possible interventionist approaches to manipulate Tregs for achieving therapeutic goals.

## Review

The current economic crisis and the fiasco at the stock market bring forth the importance of regulation. Be it Wall Street or our immune system, the indomitable presence and watchful eye of a regulator cannot be understated. The cells that perform this specialized regulator function (natural Tregs) in the immune system have gained prominence with a detailed description and characterization of their identity. We discuss in this review the role of these regulators in the context of allergic diseases and how they could be manipulated for therapeutic goals.

### Historical Perspective

The early report suggesting the presence of immune regulation dates back to 1905, when Paul Ehrlich and J. Morgenroth reported that immunization was not successful against self-tissues. Several decades later, R. E. Billingham et al. discovered the immunological basis of tolerance or immune regulation[1]. Using a murine skin model, they demonstrated that the mice immunized with allografts in utero were able to accept skin grafts from the allogenic mice [2]. It was in 1969, that Nishizuka and Sakakura, reported the first experiments that potentially indicated the presence of T cells with immuno-regulatory potentials [3]. However, a seminal observation in 1970 by Gershon et al. suggested the occurrence of immunological tolerance and attributed it to T cells [4]. But it took Sakaguchi et al. to characterize these regulatory cells as CD4^+^CD25^+ ^natural T regulatory cells. They found that athymic BALB/c mice developed autoimmune diseases, when given a population of CD4^+^CD25^+ ^T cells. Nevertheless, this phenomenon was reversed when the mice were adoptively transferred with quiescent CD4^+^CD25^+ ^T cells [5] revealing their immuno-suppressive effect. Further understanding of the constitution of T regulatory cells came from the discovery of the involvement of the forkhead box P3 (foxp3) [6], also known as transcription factor Scurfin in the regulation of immune response. The original scurfy mouse model was characterized by a mutation in the X chromosome resulting in immune dysfunction leading to splenomegaly, hepatomegaly, lymphadenopathy and early death. Interestingly, mutation of the human gene Foxp3, an ortholog of the gene mutated in scurfy mice (Foxp3) was also the cause for human immunodysregulation polyendocrinopathy enteropathy X-linked syndrome (IPEX), another sex-linked autoimmune syndrome[7]. Further studies showed that the Foxp3 was largely expressed in CD4^+^CD25^+^T cells; and retroviral transduction of conventional CD4^+ ^T cells with Foxp3 converted them to regulatory T cells with suppressive ability [6,8,9]. Thus, the current understanding is that the natural T regulatory cells are cells that possess the CD4^+^CD25^+^Foxp3^+ ^phenotype.

### Development and homeostasis of the T regulatory cell

The development of CD4^+ ^regulatory T cells occurs in the thymus from a separate lineage, but can also develop in the periphery and circulate back to thymus as reviewed by Rudensky [10]. In general, the development of hematopoietic cells initiate in the bone marrow from where the thymic lineage cells travel to the thymus. The T cells, from the double negative (DN) phenotype (CD4^-^CD8^-^) become double positive (DP) (CD4^+^CD8^+^) upon interaction of the T cell receptor (TCR) with the major histocompatibility complex (MHC) and peptides. It is in the thymic medullary region where the cells gain single positive phenotype and progress to develop either as CD4^+ ^(CD4^+^CD8^-^) or CD8^+^(CD4^-^CD8^+^) T cells respectively. The thymic medulla is also the location for the selection of CD4^+^CD25^+ ^cells [11] and commitment to Foxp3+ T regulatory cell lineage. The transcription factor Foxp3 was found to be expressed even in the DP cells, but they are turned on only in the single positive (SP) cells. The development of T regulatory cells involves a two step process as surmised by Liston et al. [12]. The first step is the encounter of the TCR with a self peptide on MHC-II, followed by exposure to B7 ligands and γc chain cytokines particularly IL-2 [10,12,13]. The induction of Foxp3 may however require CD28 ligation [14]. The T regulatory cells then make their way to the periphery to manifest their function and also are subjected to peripheral homeostatic mechanisms.

Apart from a few similarities, the homeostatic control mechanism of Tregs is different from conventional T cells. The TCR signaling is not critical for homeostatic mechanism of T regs, but it depends on three main factors as summarized by Liston et al. in their review [12]: IL-2, TGFβ and CD28 ligation. IL-2 induces the proliferation of Tregs and also contributes to their efficiency and fitness in the periphery [15]. TGFβ is needed for maintenance of Tregs, because in its absence the peripheral numbers goes down inspite of normal thymic output [16], Likewise, CD28 engagement known to be critical in the thymus seems to affect the homeostasis of Tregs in the periphery as well. Blocking of CD28 interaction with its ligands CD80 and CD86 resulted in reduced Treg numbers in the periphery [17].

### Types of Regulatory T cells

Any T cell that has the ability to suppress the immune response is known as a regulatory T cell. These cells are broadly classified as natural T regulatory cells and induced or adaptive T regulatory cells. The natural T regulatory cells (nTreg) are self antigen specific CD4^+^T cells that express CD25 in high levels and Foxp3. In addition, their phenotype is also characterized by the expression of CD62 ligand, CD103, glucocorticoid induced tumor necrosis factor receptor (GITR), Cytotoxic T-Lymphocyte Antigen 4 (CTLA-4), CD152, neurophilin and CD45RO [13,18]. The nTregs are selected in the thymus, and become regulatory T cells in the periphery.

The adaptive or induced T regulatory cells arise as a result of activation of mature T cells in the absence of optimal antigen exposure or, costimulation or in the presence of certain inhibitory cytokines. The induced Tregs include the type 1 regulatory T cells (*Tr1*) and Th3 cells. The Tr1 cells have both Th1 and Th2 phenotypic markers (chemokine receptors) like the CXCR3, CCR5, CCR3, CCR4 and CCR8. When activated they express CD40L, CD69, CD28, CTLA-4, IL-2R-α, IL-15Rα and HLA-DR. The Tr1 cells are characterized by an elevated production of IL-10, TGF-β and IL-5. Naïve CD4^+ ^T cells in the presence of immunosuppressive drugs, soluble proteins and chronic stimulation with allergic, infectious or tumor antigen develop into IL-10 producing Tr1 cells [19,20]. These cells lead to the development of a heterologous mode of suppression to non-specific antigens by the secretion of IL-10 or through mediation by CTLA-4, TGF-β, GITR or PD-1 [21]. The Th3 cells that are induced by oral antigen administration exert their suppressive activity by the production of TGFβ(reviewed in) [22].

### T cells in Allergy

Allergy is defined as an exaggerated response of the immune system to common environmental substances or 'allergens'. Coombs and Gel in 1970 classified the allergic manifestations into four different types [23]. All the four types of hypersensitivity involve either the exaggerated response of the T cells or the B cells. Most of the allergic responses involve the Th2 CD4^+ ^T cells. In the case of type-I hypersensitivity, the Th2 cells, upon coming in contact with the APC presenting an allergen, upregulates CD40 ligand on their surface which interacts with the CD40 on the B cells. This is accompanied by the production of Th2 related cytokines like IL-4, IL-5 and IL-13. A class switching of the immunoglobulins leads to the production of IgE from IgG. The IgE in turn interacts with basophils resulting in their degranulation and release of histamines, prostaglandins, chemokines and cytokines leading to smooth muscle contraction, increased vascular permeability, recruitment of more Th2 cells and eosinophils and release of neuropeptides [24-26]. The type-II and type-III hypersensitivity reactions involve complements and IgG. The type-IV hypersensitivity is mediated by antigen specific T cells, wherein the sensitized effector cells produce various cytokines including IFNγ and IL-17 and result in an inflammatory reaction.

#### Th1 and Th2 differentatiation

In 1989, Mosmann and Coffman reported that CD4^+ ^T helper cells could be divided based on their cytokine profile[27]. The Th1 cells produce IL-2, IFNγ and LTα and their development is regulated by the transcription factors T-bet and STAT-4. For their part, the Th2 cells are regulated by STAT-6, c-maf and GATA-3 and they produce cytokines such as IL-4, IL-5, IL-9 and IL-13. The Th1 type response is effective against intracellular infections and the Th2 type is required in the case of parasitic infections. Hyper activation of Th1 and Th2 type responses results in over production of cytokines leading to auto-immune disorders and allergic diseases respectively. The presence of T-regulatory cells in both scenarios prevents diseases manifestations by suppressing their activity.

#### Th17 and Treg conundrum

Implications for a major role for the cytokine IL-17, produced by a novel subset of helper cells aptly named as the Th17 have been demonstrated in the case of auto-immune diseases. IL-17 causes inflammation and induces macrophages to produce pro-inflammatory cytokines including IL-6. The cytokine IL-6 has been previously shown to inhibit the development of Tregs [28]. This novel dual function of Th17 cells, i.e. being proinflammatory and Treg suppressive, makes them an antithesis of Tregs in the context T cell mediated immune response. Given this background, the role of T cells in allergy is complex and probably tightly regulated for the right reason.

### Immune regulation by Regulatory T cells

The suppression or regulation of the immune response by T cells occurs by various pathways. This has been reviewed exhaustively and elegantly by Rouse [20]. We review here some of the mechanisms by which regulatory T cells achieve their immuno-suppressive effects. When non-professional APC present antigens to T cells and there is an absence or reduced expression of costimulatory molecules like CD80 or CD86, it results in deficient immune activation and anergy. The latter could also result in the presence of CTLA-4 on T cells. These interact with CD80 and CD86 on dendritic cells and result in the production of indoleamine 2, 3-dioxygenase (IDO), which metabolizes tryptophan and leads to a decrease in the activation of T cells [11,13,29].

The presence of a greater amount of antigen or a high degree of T cells activation could also lead to immune suppression by activation induced cell death. This process is predominantly mediated by FAS-FAS-ligand. This interaction leads to the activation of caspase enzymes which results in the degradation of chromosomal DNA and apoptotic cell death [13]. Activation induced T cell death also occurs as a result of activation of CD4^+^CD25^+^Foxp3^+ ^cells by CD3 and CD46. Activation of the Tregs upregulates the expression of granzyme A and eventually the Tregs can eradicate activated CD4^+ ^and CD8^+^T cells in the presence of perforin [13].

In the event of lower concentrations of IL-2, the suppressive function of the T cells is enhanced. This is explained by the fact that the Tregs have a higher level of IL-2 receptor expression; thereby possessing a greater ability to utilize the cytokine and deprive the other T cells of IL-2. The latter is a T cell growth factor and essential for T cell proliferation with its deficiency resulting in a hampered multiplication of the T cells. This was postulated to be the mechanism of suppression that is independent of cytokines such as IL-10 and TGF-β [25,30,31].

In patients with atopic dermatitis, there is a remarkable absence of CD4^+^CD25^+^Foxp3^+ ^Treg cells within the skin lesions, with a consequent lack of effector T cell suppression [32]. Primarily, the T-regulatory cells produce IL-10. This cytokine directly and indirectly suppresses the activity of mast cells, basophils and eosinophils [33]. IL-10 also prevents the class switching from IgG4 to IgE [24,34].

These functions are also carried out by the TGF-β secreted by the Treg cells. TGFβ interacts with fibroblasts and myofibroblasts and is known to reduce peribronchiolar extracellular matrix deposition and airway smooth muscle cell proliferation [19]. The mast cell degranulation is also suppressed by the interaction of OX40 on the Tregs with OX40 ligands on the mast cells [35]. It also prevents the secretion of IL-9 and IL-3 by the Th2 cells, thereby preventing the mucus production in the lung. The Tregs also inhibit the function of the Th1 and Th2 CD4^+^T cells and their production of cytokines in addition to suppressing the CD8^+ ^T cell activity [13]. The effect of the Tregs on these immune cells consequently results in a reduced allergic pathology caused by the immune reaction. With this knowledge, the T regulatory cells have been researched extensively to employ them as a mode of allergy prevention.

### Manipulating Tregs cells

Deficiency of Treg cells has been implicated in autoimmunity, graft rejection, and atopic diseases. Recent insights into *in vitro *and *in vivo *manipulation of these cells has led to exciting new discoveries and prospects with regards to therapeutic targeting of Treg cells to diminish the emergence or intensity of disease processes. This type of research has seen considerable advance in recent years, particularly pertaining to Foxp3+ Treg cells and IL-10-producing T cells. This review will focus on the therapeutic potential of Treg cells in allergic diseases. The basic principle behind this form of therapy resides in aiming for both natural and IL-10-secreting Tregs in order to suppress exaggerated Th2 reactions.

However, technical barriers have emerged, mainly in the form of how to promote *in vitro *proliferation of CD4^+^CD25^+ ^T cells. Godfrey et al., via stimulation of cultured cells with high doses of IL-2 and beads coated with anti-CD3 and anti-CD28, generated a 100-fold expansion in cell number while retaining a strong suppressive function [36]. An even more impressive result was obtained with high-dose of IL-2 and artificial antigen-presenting cells (for repeated stimulation through CD3 and CD28), achieving a 40,000-fold increase in cell counts while retaining the expression of CD25 and lymph node homing receptors and displaying a greater suppressive power than freshly isolated CD4^+^CD25^+^T cells [37]. It is important to emphasize that antigen specificity of Tregs should be carefully controlled in order to avoid development of auto-reactive effector T cells or Tregs possessing erroneous antigenic specificity [33].

One of the potential targets for development of therapeutic agents is the transcription factor, shown to be selectively expressed in natural Tregs. In addition, retroviral gene transfer of Foxp3 causes naïve T cells to display a regulatory T cell phenotype similar to that of natural Tregs [6]. Foxp3 is the ultimate regulator of Treg expression, and hence has a vital role in promoting immune suppression through Treg activity, as shown in a murine model in which a fatal autoimmune syndrome was avoided in null mice by ectopic expression of and/or transfer of CD4^+^CD25^+^T cells into these subjects [8]. This highlights the potential use of induction to enhance suppression of the Th2 induced allergic response. Currently various strategies are adopted to up-regulate the Treg cells in conditions of allergy, transplantation and autoimmune diseases.

#### Immunotherapy

The main principle behind immunotherapy in atopic patients is to increase the number of Tregs, well known to diminish the pulmonary inflammatory response to air-borne allergens, as shown in murine models by both Treg depletion and adoptive transfer of Tregs [38,39]. Subcutaneous immunotherapy in asthmatics has already been in use, but titrating the dosing and treatment schedules remains a challenge [40]. Utilizing specific subcutaneous immunotherapy enhances Treg proliferation and production of IL-10 [41], and development of specific T cell peptide-based immunotherapy is currently under way [42].

IL-2 has been shown to have a critical role in thymic development, expansion within peripheral blood, and the suppressive activity of CD4^+^CD25^+^Foxp3^+ ^Tregs [43,44], although the exact mechanism behind its influence on these cells is still under study. Blockade of the IL-2 receptor pathway in peripheral Tregs led to decreased suppressive function and slower growth [45] as well as a greater predisposition to development of autoimmune disease [46]. As an attractive candidate for *in vivo *therapy, a number of studies have surfaced in which cytokine manipulation was explored, demonstrating the positive effect of recombinant IL-2 (rIL-2) on Treg expansion [44,47]. However, this observation has been challenged by a recently published article by Wilson et al., in which worsening of airway inflammation was observed in murine subjects treated with rIL-2 [48]. In contrast, when IL2:anti-IL-2 complexes were administered both before and after allergenic stimulation, a remarkable increase in Treg counts was observed along with an increase in mRNA and IL-10 levels in lung tissue; in addition, subjects displayed decreased pulmonary pathologic findings (mucus secretion, airway inflammation and hyper-responsiveness), corroborating a similar report by Boyman et al. [49]. The IL2:anti-IL-2 complex has been shown to cause enhanced secretion of IL-35, a cytokine which expands Tregs and augments their secretion of IL-10 [50], an important mediator in controlling airway inflammation [51]. The reasons for these findings are not entirely clear, but several hypotheses can be postulated. Even though the current thought is that IL-2 contributes more heavily towards immune tolerance, T effector cells (Teff), which have a proinflammatory action, can also be activated by IL-2 under certain conditions [52], and a preferential Teff cell activation may have taken place in the subjects presenting worsening symptoms when exposed to rIL-2. Interestingly, complexing IL-2 with an antibody stabilizes the cytokine and increases its half-life; the reported effects of this on T cell populations is not uniform, as studies have shown that preferential CD4^+ ^or CD8^+ ^T cells can occur depending on which anti-IL-2 clone is utilized [48,53,54].

In a mouse model, splenic DCs have been targeted to induce the Foxp3+ Treg cells. Monoclonal antibodies to the CD11c^+^DEC205^+^CD8^+ ^subtype of DC resulted in reduced ability of these cells to present antigen to the CD4 T cells [55]. This repeated stimulation of T cells with immature DCs resulted in elevated IL-10 production. Furthermore, it suppresses the cytokine producing ability of the CD8+ T cells. TRAIL (TNF-related apoptosis inducing ligand) is another molecule reported to expand CD4^+^CD25^+^T cells in mouse models.

#### Steroids

There is a large volume of evidence suggesting that CD4^+^CD25^+^T cells have an active role in suppressing the Th2 response seen in allergic reactions, and that patients with deficient cell counts would be overwhelmed by exposure to allergens and thus unable to adequately harness their immune response. Ling et al. demonstrated that, when compared with non-atopic controls, CD4^+^CD25+ cells of atopic patients have an impaired ability to suppress proliferation and IL-5 release by CD4^+^CD25^-^T cells, particularly in patients with hay fever assessed during the pollen season [56]. However, upon removal of CD4^+^CD25^+^T cells from the blood of non-atopic patients, exposure to allergens led to a Th2 cytokine profile comparable to atopics [57]. A study by Hartl et al. compared several cell and chemokine parameters in asthmatic and healthy children. While peripheral blood analysis failed to reveal a difference in absolute numbers of CD4^+^CD25^+^T cells, bronchoalveolar lavage fluid of asthmatic children possessed significantly diminished levels of CD4^+^CD25^+^T cells and IL-10 and TGF-β mRNA [58]. These same markers were found to be higher in asthmatic patients using inhaled corticosteroids when compared with patients not on this form of therapy [58].

Corticosteroids are known to actively suppress the Th2 response, and mounting evidence has shown that this activity is derived from their stimulatory effect on Tregs, inducing the latter to augment secretion of IL-10 and expression of Foxp3 [59,60], while administration of anti-IL-10 antibody reversed the steroid-induced suppression. In addition, T cells in corticosteroid-resistant asthmatics have diminished IL-10 secreting capacity as compared to corticosteroid-sensitive subjects [61], reinforcing the importance of this activity in the clinical efficacy of steroids. Peek et al. demonstrated that combining an inhaled corticosteroid with an inhaled beta-2 agonist (namely, salmeterol) had an additive effect in promoting IL-10 secretion and blocking the release of the Th2 cytokines IL-5 and IL-13 [62]. However, this inability to secrete IL-10 is reversible, as demonstrated in a study in which steroid-resistant asthmatics who received calcitriol (0.5 mcg per day for 7 days) in addition to dexamethasone presented IL-10 levels comparable to steroid-sensitive subjects, establishing vitamin D3 as an important ancillary agent in restoring the IL-10 secreting capacity of Tregs [63]. Consequently, IL-10 acts to increase expression of the glucocorticoid receptor (previously downregulated by the presence of dexamethasone). Incidentally, glucocorticoids are known to favor Treg proliferation while limiting that of Teff, and their use as adjuvant therapy along with IL-2 has shown promise, supported by a study in which 300,000 IU of IL-2 daily in combination with dexamethasone significantly expanded CD4^+^CD25^+ ^Tregs [47].

#### PAMPs

PAMPS (or pathogen associated molecular patterns) are the molecular signature of microbial infections. PAMPs include various microbial components such as the glycoproteins, lipoproteins, DNA, RNA, etc. These biomolecules interact with pattern recognition receptors (PRR) on host immune cells and potentiate an innate immune response and subsequently the adaptive response. One of the PRRs in the mammalian system is the toll like receptors (TLR). Studies have shown that Treg function could be augmented through their engagement of TLRs. Acting via TLR-2 and TLR-5, respectively, heat-shock protein 60 (HSP60) and flagellin enhance the suppressive functions of CD4^+^CD25^+ ^T cells and, in the case of flagellin, the expression of Foxp3 was also enhanced [64-66]. Tregs treated with HSP60 promoted T cell inhibition through both direct cell contact and production of IL-10 and TGFβ.

Our studies in mice have shown that constant stimulation with Herpes simplex virus-1 for more than 9 months in C57BL/6 mice increased the frequency of CD4^+ ^CD25^+^Foxp3^+ ^cells in their spleen and regional lymph nodes (Nandakumar and Kumaraguru, Unpublished). HSV is known to engage TLR-2, 3 and 9 and upregulate these TLRs on dendritic cells and CD8^+ ^T cells. The constant antigenic stimulation reduced the CD8^+ ^T cell function, in addition to upregulating the frequency of regulatory T cells.

In a murine asthma model, pre-treatment with *Mycobacterium vaccae *resulted in reducing the lung histopathology [67,68]. Other microbial agents aiding the induction of regulatory T cells included measles virus and hepatitis C virus [69,70]. Though TLR ligands are promising targets for future Treg-directed therapy, it is important to study the dosage schedule and timing of the PAMP stimulation needed to obtain the required outcome. Incidentally, microbial exposure pattern during childhood may be a determining factor in the allergy outcomes in adults. This has been one of the focuses of our laboratory currently. We plan to expand the studies to analyze the impact of neonatal microbial exposure on the development of allergies in Western world compared to developing or underdeveloped countries. Collectively, these are studies attempting to prove the popular "hygiene hypothesis".

#### Immunosuppressive Agents

Rapamycin is an immunosuppressive agent used in allograft rejection prevention; it has recently been shown that this drug can selectively expand CD4+CD25+Foxp3+ T cells in mice, potentially serving as a therapeutic option in T-cell diseases [71]. Rapamycin also expanded the percentage of suppressive CD8+ T cells, especially those expressing CD103 [72]. Another immunosuppressive agent fingolimod (FTY270), a sphingosine-1-phosphate receptor 1 modulator, expanded antigen specific regulatory T cells. FTY270 exhibited the ability to convert Foxp3 negative cells to Foxp3 positive cells [73].

The role of estrogen in maintaining immune tolerance in normal individuals has been well described, as well as its importance in preventing autoimmune disease. Even at physiological levels, estradiol has been shown to increase levels of CD4+CD25+ T cells, Foxp3 expression, and IL-10 expression [74,75]. As CD4+CD25+ T cells are paramount for development of fetal tolerance during pregnancy, the higher circulating levels of estrogen during gestation represent an adaptive mechanism to increase Treg expression.

#### Exercise

Benefits of regular exercise in the management of allergy and asthma are ample. Yeh et al. reported that regular tai chi exercise induced the mobility and function of T regulatory cells. The frequency of CD4^+^CD25^+^T cells producing TGF-β and IL-10 increased almost by two fold upon regular exercise [76]. These results also correlated with our work, where individuals performing regular anaerobic exercises like lifting weights had significantly higher levels of regulatory T cells. The CD4^+^CD25^+^Foxp3^+^T cells in regular anaerobic exercisers were three times greater compared to the control group (unpublished).

### Parting Thoughts

With the existing knowledge on T regulatory cells, several interventional techniques can be employed to modulate the development and function of these cells. The exploitation of Tregs is essential in circumstances like allergy, autoimmunity and transplantation. Although the most used method of Treg frequency enhancement is the use of steroids, the use of these biocomponents is loaded with various side effects. Current research has emphasized the ability of certain other factors for improving Treg levels. These include regular exercise and constant PAMP stimulation, which could be attained by the use of various probiotics or prebiotics.

## Competing interests

The authors declare that they have no competing interests.

## Authors' contributions

SN, Post Doctoral Research Associate, works on the role of Tregs in Exercise, Chronic Infections and neonatal microbial exposure studies. CWTM, Resident, Division of Allergy and Immunology, Dept. of Internal Medicine, is the clinical collaborator and contributed towards the therapeutic potentials of Tregs. UK, Immunology Faculty in the Department of Microbiology. He has been working on regulatory T cells and is the Supervisor and Mentor for Dr. Nandakumar.
